# Prognostic Factors in Patients With Spontaneous Pneumothorax Treated With Video-Assisted Thoracoscopy

**DOI:** 10.1155/DTE.2.1

**Published:** 1995

**Authors:** Franz M. N. H. Schramel, Tom G. Sutedja, Julius P. Janssen, Miguel A. Cuesta, Johan C. van Mourik, Pieter E. Postmus

**Affiliations:** 1 Department of pulmonary medicine Free University Hospital PO Box 7057 Amsterdam 1007 MB The Netherlands; 2 Department of surgery Free University Hospital Amsterdam The Netherlands

## Abstract

To analyse the efficacy of video-assisted thoracoscopy (VAT) in patients with spontaneous pneumothorax (SP) and to identify clinical factors associated with outcome after VAT, one hundred and one VATs were performed in 97 patients in this prospective study. Based on thoracoscopic appearance of the visceral pleura three groups were identified, group 1 (n = 23) showing no abnormalities treated with talcage, group 2 (n = 23) showing bullae less than 2 cm treated with talcage and coagulation, and group 3 (n = 51) showing bullae larger than 2 cm treated with bullectomy by staplers, coagulation and pleural scarification. Data were analyzed with regard to clinical factors such as age, smoking behavior, pulmonary function and recurrent pneumothorax at presentation. No perioperative deaths occurred. Overall relapse rate was 4.0% during a follow-up period of 3 to 38 months (median 27.2). Univariate analysis did not show any association of clinical factors with postoperative complications (n = 19). Patients with extensive pulmonary lesions had longer drainage and hospitalization time, probably due to insufficient sealing effects of stapler devices and/or pleural scarification. Using multivariate analysis, none of the clinical factors had any association with complication rate, drainage/hospitalization time or relapses after VAT. Only patients with bullae larger than 2 cm treated with bullectomy by stapler devices were associated with longer drainage and hospitalization time. This study suggests that VAT is effective in the treatment of spontaneous pneumothorax, although the use of stapler devices and/or pleural scarification was associated with longer drainage and hospitalization time, however, none of the clinical factors were associated with the outcome after VAT.


*Diagnostic and Therapeutic Endoscopy*, 1995, Vol. 2, pp. 1–5
Reprints available directly from the publisher
Photocopying permitted by license only

© 1995 Harwood Academic Publishers GmbH
Printed in Singapore

### Prognostic Factors in Patients With Spontaneous Pneumothorax Treated With Video-Assisted Thoracoscopy

FRANZ M. N. H. SCHRAMEL,* TOM G. SUTEDJA,* JULIUS P. JANSSEN,* MIGUEL A. CUESTA,@ JOHAN C. VAN MOURIK@ and PIETER E. POSTMUS*

*From the Departments of pulmonary medicine* and surgery,@ Free University Hospital, Amsterdam, the Netherlands*

*(Received October 31, 1994; revised March 6, 1995)*

To analyse the efficacy of video-assisted thoracoscopy (VAT) in patients with spontaneous pneumothorax (SP) and to identify clinical factors associated with outcome after VAT, one hundred and one VATs were performed in 97 patients in this prospective study. Based on thoracoscopic appearance of the visceral pleura three groups were identified, group 1 (n = 23) showing no abnormalities treated with talcage, group 2 (n = 23) showing bullae less than 2 cm treated with talcage and coagulation, and group 3 (n = 51) showing bullae larger than 2 cm treated with bullectomy by staplers, coagulation and pleural scarification. Data were analyzed with regard to clinical factors such as age, smoking behavior, pulmonary function and recurrent pneumothorax at presentation. No perioperative deaths occurred. Overall relapse rate was 4.0% during a follow-up period of 3 to 38 months (median 27.2). Univariate analysis did not show any association of clinical factors with postoperative complications (n = 19). Patients with extensive pulmonary lesions had longer drainage and hospitalization time, probably due to insufficient sealing effects of stapler devices and/or pleural scarification. Using multivariate analysis, none of the clinical factors had any association with complication rate, drainage/hospitalization time or relapses after VAT. Only patients with bullae larger than 2 cm treated with bullectomy by stapler devices were associated with longer drainage and hospitalization time. This study suggests that VAT is effective in the treatment of spontaneous pneumothorax, although the use of stapler devices and/or pleural scarification was associated with longer drainage and hospitalization time, however, none of the clinical factors were associated with the outcome after VAT.

**KEY WORDS:** Video-assisted thoracoscopy, spontaneous pneumothorax

### INTRODUCTION

Recurrence of a primary spontaneous pneumothorax (SP) is generally considered to be an indication for surgical treatment.[1–3] The main purpose of surgery is to achieve effective pleurodesis and to treat causal pulmonary lesions. In the past, chemical pleurodesis was performed by tube thoracostomy or thoracoscopy; bullectomy and pleurectomy by thoracotomy. Recently, video-assisted thoracoscopy (VAT) made bullectomy possible by means of less invasive procedures due to improved endoscopic instrumentation and video imaging techniques.[4–10] Pleural symphysis can be achieved chemically using tetracycline, doxycycline or talc,[8,11,12] mechanically by pleural abrasion,[13,14] thermally using electrocoagulation/Nd-YAG laser,[5,15] or surgically by stripping the apical part of the parietal pleura with artery grasping forceps.[7,10,16] Relapse rates of bullectomy with endoscopic stapler devices vary from 0 to 13%.[3,17,22]

The aims of this prospective study are: 1) to analyse the efficacy of VAT in patients with spontaneous pneumothorax and 2) to identify clinical factors associated with outcome after VAT.

### MATERIALS AND METHODS

### Patients

From December 1991 to December 1994, 101 VATs were performed in 97 patients with SP. There were 74 male and 23 female patients, mean age 33.1 years, ranging from 16 to 83 years. All patients had a negative history of chronic

Address for correspondence: Dr. F. Schramel, pulmonologist, MD., Department of Pulmonary Medicine, Free University Hospital, PO Box 7057, 1007 MB Amsterdam.

obstructive pulmonary disease (COPD) at the time of presentation. The characteristics of the patients are given in Table 1.

### Technique

All patients underwent VAT under general anesthesia, with double lumen intubation in the standard thoracotomy position. Initial trocar placement was in the fifth or sixth intercostal space in which the thoracoscope is inserted into the pleural cavity. A second smaller trocar is inserted in the second intercostal space in the midclavicular line for insertion of the grasping forceps. Complete collapse of the lung was obtained in all cases. The visceral pleura was judged as being normal or showing blebs and/or bullae.

The respective treatments were: group 1, insufflation with 3 grams talc if the visceral pleura was normal. Group 2, coagulation by electrocautery if blebs or bullae less than 2 cm were seen, followed by talc poudrage. Group 3, stapling with Autosuture Multifire EndoGIA® 3.5, introduced through a third trocar in the third or fourth intercostal space in the posterior axillary line for bullae larger than 2 cm. In this last group the parietal pleura was also scarified using electrocoagulation at the level of the first to the fifth costae.

A chest tube (charierre 20) was inserted under videothoracoscopic guidance through the fifth or sixth intercostal space in the midclavicular line on the ventral side of the pleural cavity. Vacuum drainage was performed until air leakage ceased and the lung had completely reexpanded. The chest tube was removed if the lung remained expanded one day after clamping.

Drainage time (DT) of more than four days was considered prolonged. Total days of admission (hospitalization time (HT)) after VAT was considered prolonged if this exceeded five days.

Six weeks after VAT, a pulmonary function test was performed by spirometry. Forced expiratory volume in one second and vital capacity were measured using a pneumotachometer (Jaeger, Masterlab). Normal predictive values were taken from Quanjer *et al.*[18]

**Table 1** Patient characteristics

| | *Group 1* *n = 23* | *Group 2* *n = 23* | *Group 3* *n = 51* | *Total* *n = 97* |
|---|---|---|---|---|
| Mean age ± SD | 26.1 ± 5.6 | 28.3 ± 9.9 | 38.5 ± 15.7 | 33.1 ± 13.8 |
| Smokers | 17 | 17 | 33 | 67 |
| Obstructive PF* | 3 | 2 | 7 | 12 |
| Recurrent SP | 8 | 7 | 11 | 26 |

Abbreviations: n: number, SD: standard deviation, PF: pulmonary function, SP: spontaneous pneumothorax.
*: Pulmonary function tests of 15 patients were not available.

### Statistics

Univariate analysis: the unpaired t test was used to assess differences of the various patient characteristics and for comparing DT and HT in different subgroups. Chi-square test was used for comparing the complication rate in different subgroups.

Multivariate analysis (logistic regression) was used to determine the significant association of several clinical factors (age, smoking behavior, pulmonary function, recurrent SP, endoscopic appearance) and ultimate result (complication rate, drainage time, hospitalization time, relapse) after VAT. A p value of $< 0.05$ was considered statistically significant.

### RESULTS

### Patient and Treatment Data

One hundred and one VATs were performed in 97 patients for SP. Patient characteristics are described in Table 1. Four patients underwent VAT twice, due to contralateral pneumothorax. In three patients of group 3, a submammarian incision was needed to remove large bullous degenerated lung tissue.

Data were analyzed in regard to clinical factors such as age, smoking behavior, pulmonary function, first or recurrent pneumothorax at presentation and thoracoscopic appearance. No significant differences in smoking behavior, pulmonary function, first or recurrent SP at presentation between the three groups was found. Patients in group 3 were significantly older ($p < 0.001$) even if patients with a obstructive pulmonary function were excluded from analysis. There was no significant difference in the three groups regarding treatment indications for first or recurrent pneumothoraces. Followup ranged from 3 to 38 months (median 27.2).

### Complications

No complications during the 101 VAT procedures and no perioperative deaths occurred. Nineteen patients (19%) had minor postoperative complications, fifteen of them of pulmonary origin (Table 2). Invasive interventions were not required except the insertion of new chest tubes in five patients. Conversion to open thoracotomy was not necessary. Four patients had relapse of the pneumothorax (4.0%), all from group 3. The relapse rate in this group was 7.7%, occurring within the first two years (ranging from 1.7 to 22.5 months, median 3.3).

The complication rates among the three groups were not significantly different, also when several clinical factors were analyzed separately (Table 3).

### Drainage and Hospitalization Time

DT and HT are given in Table 4. Significantly shorter drainage and hospitalization times were seen in patients of group 2, patients without or with bullae smaller than 2 cm and patients with an uncomplicated postoperative course. Patients with obstructive pulmonary function had a shorter DT but a longer HT. Non-smokers and patients treated with coagulation showed a tendency toward a shorter DT. Patients presenting with a first pneumothorax had a shorter HT.

### Multivariate analysis

Multivariate analysis (logistic regression) of the clinical factors (age, smoking behavior, pulmonary function, recurrent pneumothorax at presentation) showed that only the thoracoscopic demonstration of bullae larger than 2 cm was significantly associated with a prolonged DT and HT. Other clinical factors were not associated with postoperative complications, prolonged drainage/hospitalization time or relapses.

**Table 2** Postoperative complications

| | *n* |
|---|---|
| Relapse | 4 |
| Redrainage | 5 |
| Atelectasis | 2 |
| Pneumonia | 2 |
| Reexpansion edema | 2 |
| Other | 4 |
| Total | 19 |

Abbreviation: n: number.

**Table 3** Postoperative complications and clinical factors

| | *No complications* *n = 82* | *Complications* *n = 19* | *p* |
|---|---|---|---|
| Mean age ± SD | 32.5 ± 13.5 | 33.9 ± 14.9 | 0.68[1] |
| Age > 50 years | 10 | 4 | 0.25[2] |
| Group 1/2/3 | 21/21/40 | 4/4/11 | 0.74[2] |
| Smoking | 58 | 13 | 0.84[2] |
| Obstructive PF* | 8 | 4 | 0.19[2] |
| Recurrent SP | 20 | 7 | 0.27[2] |

Abbreviations: n: number, p: significance, SD: standard deviation, >: larger than, PF: pulmonary function, SP: spontaneous pneumothorax.
*: Pulmonary function tests of 15 patients were not available.
[1]: Unpaired t test, [2]: Chi-square test.

### DISCUSSION

There is no consensus regarding the best management of SP. Treatment is aimed at preventing relapses. Surgery provides the lowest relapse rates, varying from 0% to 3.6%.[20,21] However, treatment morbidity is high, in contrast to tube thoracostomy, with a relapse rate up to 57%.[20] VAT has proven its efficacy, with low relapse rates varying from 0 to 13% and low morbidity comparing to conventional thoracotomy.[3,17]

In our series of 97 patients 101 VATs were successful with low morbidity, no mortality and a relapse rate of 4.0% at a median follow-up of more than two years. Unlike the

**Table 4** Mean drainage and hospitalization time (days)

| | *DT* ± *SD* | *p*[1] | *HT* ± *SD* | *p*[1] |
|---|---|---|---|---|
| Group 1 | 4.6 ± 2.8 | < 0.001 | 6.0 ± 2.8 | 0.003 |
| Group 2 | 3.3 ± 0.5 | | 5.0 ± 0.9 | |
| Group 3 | 4.9 ± 3.0 | | 6.9 ± 4.3 | |
| Age < 50 years | 4.5 ± 2.7 | 0.11 | 6.0 ± 2.7 | 0.05 |
| Age > 50 years | 4.0 ± 1.5 | | 7.1 ± 6.5 | |
| Non-smokers | 3.9 ± 1.8 | 0.08 | 6.3 ± 4.7 | 0.30 |
| Smokers | 4.6 ± 2.8 | | 6.1 ± 2.8 | |
| Normal PF* | 4.8 ± 2.9 | 0.01 | 6.1 ± 2.9 | 0.05 |
| Obstructive PF* | 3.5 ± 0.7 | | 7.7 ± 6.9 | |
| First SP | 4.3 ± 2.4 | 0.10 | 5.9 ± 2.5 | 0.004 |
| Recurrent SP | 4.8 ± 3.0 | | 7.1 ± 5.3 | |
| No bullae/bullae < 2 cm | 3.9 ± 2.0 | 0.02 | 5.9 ± 2.1 | 0.005 |
| Bullae > 2 cm | 4.9 ± 3.0 | | 7.0 ± 4.3 | |
| Complication – | 4.1 ± 2.3 | 0.01 | 5.6 ± 2.4 | 0.001 |
| Complication + | 5.6 ± 3.3 | | 8.5 ± 5.9 | |
| Coagulation – | 4.6 ± 2.7 | 0.08 | 6.1 ± 2.7 | 0.51 |
| Coagulation + | 4.0 ± 2.3 | | 6.3 ± 4.5 | |
| Total | 4.4 ± 2.6 | | 6.2 ± 3.5 | |

Abbreviations: DT: drainage time, HT: hospitalization time, SD: standard deviation, p: significance, >: larger than, < less than, PF: pulmonary function, SP: spontaneous pneumothorax.
*: Pulmonary function tests in 15 patients were not available.
[1]: Unpaired t test.

**Table 5** Significance (p[1]) of association between clinical factors and outcome after VAT, using logistic regression analysis.

| | *Complications* | *DT* | *HT* | *Relapse* |
|---|---|---|---|---|
| Age | 0.47 | 0.25 | 0.13 | 0.57 |
| Smoking | 0.85 | 0.28 | 0.70 | 0.96 |
| Obstructive PF* | 0.31 | 0.24 | 0.53 | 0.81 |
| Recurrent SP | 0.25 | 0.44 | 0.93 | 0.59 |
| Bullae > 2 cm | 0.30 | 0.005 | 0.01 | 0.87 |

Abbrevations: DT: drainage time, HT: hospitalization time, p: significance, SP: spontaneous pneumothorax, PF: pulmonary function, >: larger than.
*: Pulmonary function tests in 15 patients were not available.
[1]: Logistic regression analysis.

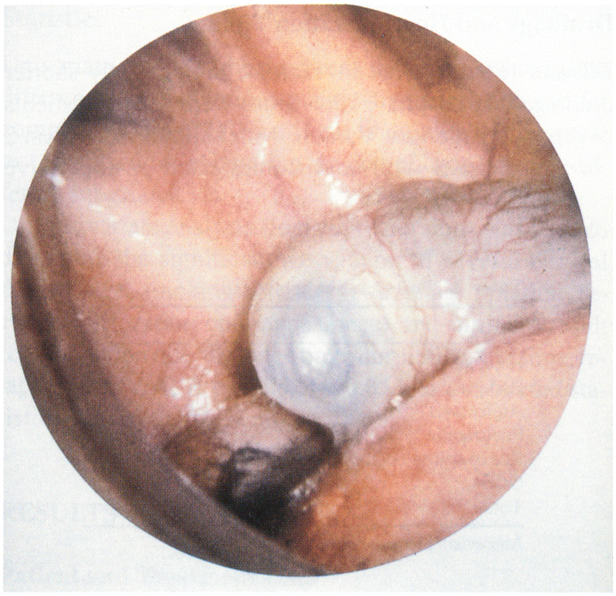

**Figure 1** Endoscopic findings in a patient of group 3.

classification of Vanderscheuren, we distinguished only three groups.[19] None of the patients without pulmonary lesions showed adhesions. In the group with bullae larger than 2 cm the relapse rate was 7.7%. This rate is comparable with previous publications.[3,22] Nineteen patients had minor postoperative complications, of which 79% were of pulmonary origin. Using univariate analysis we did not find a clinical factor associated with a complicated postoperative course. Even factors such as smoking, age and pulmonary function, which presumably are risk factors for a complicated course after surgery,[23] were not significantly related to postoperative complication. This was probably due to less postoperative pain and rapid mobilization after VAT.

Analyzing the patients characteristics, we demonstrated that patients with extensive pulmonary lesions were significantly older. The relation between age and parenchymal changes is not surprising as it is known that bullae grow with age.[24] Assuming that all parenchymal abnormalities were adequately treated, the prolonged drainage time in patients with large bullae might not be due to the bullae themselves but to an insufficient sealing effect of the staplers or inadequate pleurodesis by pleural scarification. This is supported by the fact that patients treated with coagulation had shorter drainage times. The additional sealing effect of electrocautery has been previously described.[15]

It was also surprising that patients with obstructive pulmonary disease had shorter drainage times. However, these patients were equally divided among the three groups. Thus the presence of bullae was not correlated with obstructive pulmonary function testing in patients with spontaneous pneumothorax. This explains why these patients did not show prolonged drainage time.

Using multivariate analysis age, smoking behavior, pulmonary function and recurrent pneumothorax at presentation were not associated with clinical outcome after VAT. Only the presence of large bullae had effect on drainage and hospitalization time, probably related to the treatment modality.

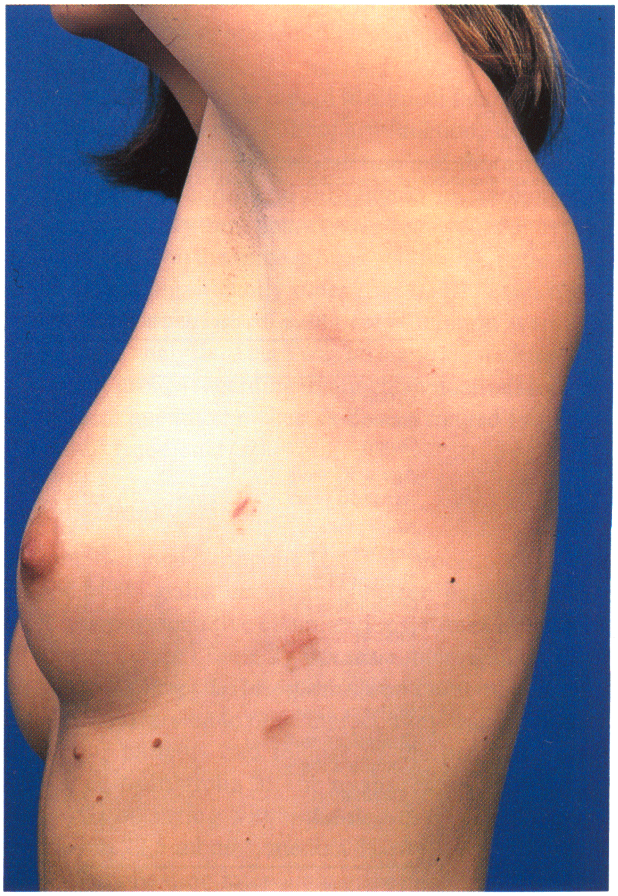

**Figure 2** Cosmetic result after VAT in a 19-year-old female patient.

In conclusion, VAT is an effective technique to treat spontaneous pneumothorax, although the usage of stapler devices and/or pleural scarification was associated with longer drainage and hospitalization time. There are no clinical factors such as age, smoking behavior, pulmonary function or presentation with recurrent pneumothorax which are associated with the outcome after VAT.

### REFERENCES

1. Light RW. Pneumothorax. In: Pleural diseases. Second edition. Lea & Febiger. Philadelphia, London, 1990;237–262.
2. Loddenkemper R, Boutin C. Thoracoscopy: present diagnostic and therapeutic indications. Eur Respir J, 1993;6:1544–1555.
3. Kaiser LR. Video-assisted thoracic surgery. Current state of the art. Ann Surg, 1994;220:720–734.
4. Landreneau RJ, Mack MJ, Hazelrigg RS, Dowling RD, Acuff TE, Magee MJ, Ferson PF. Video-assisted thoracic surgery: Basic technical concepts and intercostal approach strategies. Ann Thorac Surg, 1992;54:800–807.
5. Melvin WS, Krasna MJ, McLaughlin JS. Thoracoscopic management of spontaneous pneumothorax. Chest, 1992;102:1875–1876.
6. Bagnato JV. Treatment of recurrent spontaneous pneumothorax. Surg Laparoscopy and Endoscopy, 1992;2:100–103.
7. Inderbitzi R, Furrer M. The surgical treatment of spontaneous pneumothorax by video-thoracoscopy. J Thorac Cardiovasc Surg, 1992;40:330–333.
8. Mack MJ, Aronoff RJ, Acuff TE, Douhit MB, Bowman RT, Ryan WH. Present role of thoracoscopy in the diagnosis and treatment of diseases of the chest. Ann Thorac Surg, 1992;54:403–409.
9. Janssen JP, v Mourik J, Cuesta Valentin M, Sutedja G, Gigengack K, Postmus PE. Treatment of patients with spontaneous pneumothorax during videothorascopy. Eur Respir J, 1994;7:1281–1284.
10. Nathanson LK, Shimi SM, Wood RAB, Cuschieri A. Videothoracoscopic ligation of bulla and pleurectomy for spontaneous pneumothorax. Ann Thorac Surg, 1991;52:316–319.
11. Bresticker MA, Oba J, LoCicero J, Greene R. Optimal pleurodesis: A comparison study. Ann Thorac Surg, 1993;55:364–367.
12. Almind M, Lange P, Viskum K. Spontaneous pneumothorax: Comparison of simple drainage, talc pleurodesis, and tetracylcine pleurodesis. Thorax, 1989;44:627–630.
13. Nkere UU, Griffin SC, Fountain SW. Pleural abrasion: A new method of pleurodesis. Thorax, 1991;46:596–598.
14. Krasnik M, Stimpel H, ???. Treatment of primary spontaneous pneumothorax with intrapleural tetracycline instillation or thoracotomy. Scand J Thor Cardiovasc Surg, 1993;27:49–51.
15. Moghissi K, Dench M, Neville E. Effect of the non-contact mode of Yag laser on pulmonary tissues and its comparison with electrodiathermy: An anatomico-pathological study. Lasers in Med Sci, 1989;4:17–23.
16. Donnelly RJ, Page RD, Cowen ME. Endoscopy assisted microthoracotomy: Initial experience. Thorax, 1992;47:490–493.
17. Hazelrigg SR, Landreneau RJ, Mack M, Acuff T, Seifert PE, Auer JE, Magee M. Thoracoscopic stapled resection for spontaneous pneumothorax. J Thorac Cardiovasc Surg, 1993;105:389–393.
18. Quanjer H, Tammeling GJ, Cotes JE, Pedersen OF, Peslin R, Yernault JC. Lung volumes and forced ventilatory flows. Report working party standarization of lung function test European community for steel and coal. Official statement of the European Respiratory Society. Eur Respir J, 1993;6s:5–40.
19. Vanderscheuren RG. Le talcage pleural dans le pneumothorax spontané. Poumon-Coeur 1981;37:273–276.
20. Gobel WG, Rhea WG, Nelson IA, Daniel RA. Spontaneous pneumothorax. J Thorac Cardiovasc Surg, 1963;46:331–345.
21. Donahue DM, Wright CD, Viali G, Mathisen DJ. Resection of pulmonary blebs and pleurodesis for spontaneous pneumothorax. Chest, 1993;104:1767–1769.
22. Inderbitzi R, Leiser A, Furrer M, Althaus U. Three years' experience in video-assisted thoracic surgery (VATS) for spontaneous pneumothorax. J Thorac Cardiovasc Surg, 1994;107:1410–1415.
23. Merli GJ, Weitz H. Approaching the surgical patient. The role of the medical consultant. Clin Chest Med, 1993;14:205–210.
24. Boutin C. Thoracoscopic findings in spontaneous pneumothorax. In: Boutin C, Viallat JR, Aeloney Y. Practical thoracoscopy, Berlin, Springer Verlag, 1991, 73–81.

